# Correction to: A comparative study of dementia knowledge, attitudes and care approach among Chinese nursing and medical students

**DOI:** 10.1186/s12909-020-02398-6

**Published:** 2020-11-26

**Authors:** Yao Wang, Lily Dongxia Xiao, Rong Huang

**Affiliations:** 1 Xiangya School of Nursing, Central South University, Changsha, Hunan Province China; 2 College of Nursing and Health Sciences, Flinders University, Adelaide, South Australia Australia

**Correction to: BMC Med Educ 20, 436 (2020)**

**https://doi.org/10.1186/s12909-020-02365-1**

Following publication of the original article [1], we have been informed that the author Rong Huang was incorrectly affiliated to “College of Nursing and Health Sciences, Flinders University, Adelaide, South Australia, Australia”

The correct affiliation is:

Xiangya School of Nursing, Central South University, Changsha, Hunan Province, China

The original article has been corrected.
