# Supplementary material for: A comparative study of dementia knowledge, attitudes and care approach among Chinese nursing and medical students
Source: BMC Med Educ. 2020 Nov 16;20:436. doi: 10.1186/s12909-020-02365-1 (PMC7670709; doi:10.1186/s12909-020-02365-1)
Supplement: Supplementary file 3 — Additional file 3. [file 12909_2020_2365_MOESM3_ESM.docx]

**A comparative study of dementia knowledge, attitudes and care approach among Chinese nursing and medical students**

Yao Wang^1§^ Lily Dongxia Xiao^1,2§^, Rong Huang

^1^Xiangya School of Nursing, Central South University, Changsha, Hunan Province, China

^2^College of Nursing and Health Sciences, Flinders University, Adelaide, South Australia, Australia

§Corresponding author

Email addresses:

YW: sweet0131@163.com

LDX: [lily.xiao@flinders.edu.au](mailto:lily.xiao@flinders.edu.au)

RH: huangrong@163.com

**Abstract**

**Background**

Dementia care requires inter-disciplinary collaboration starting from formal health professional education. Yet, little is known about how undergraduate medical and nursing students perceive dementia care in China. The aim of this study was to investigate undergraduate medical and nursing students’ dementia knowledge, attitudes and care approach in China.

**Methods**

A cross-sectional survey was conducted. Students enrolled in a 5-year Bachelor of Medicine Program and a 4-year Bachelor of Nursing Program from four universities with campuses across Eastern, Western, Southern and Northern China were recruited into the study. Three validated instruments, Alzheimer’s Disease Knowledge Scale (ADKS), Dementia Care Attitude Scale (DCAS) and Approach to Advanced Dementia Care Questionnaire (ADCQ), were used to examine students’ dementia knowledge, attitudes and perceived care approach. Data were collected using a self-administered survey.

**Results**

The number of medical and nursing students completing the survey was 526 and 467 respectively. Students’ overall knowledge about dementia was poor, but attitudes were generally positive. The overall mean score of students’ dementia knowledge examined by the ADKS was 19.49 (SD=2.82) out of 30, students’ attitudes to dementia was 29.92(SD=3.35) out of 40, and students’ person-centred care approach of dementia was 5.42 (SD=2.20) out of 13. Medical students demonstrated higher dementia knowledge scores and showed less positive attitude scores than nursing students (p<0.05). Students would not apply a person-centred care approach. There were no statistically significant differences in the mean scores of ADCQ between nursing students and medical students.

**Conclusions**

Study results highlight the urgent need to implement an inter-disciplinary approach to increasing dementia education among Chinese medical and nursing students, and ensuring that students have adequate knowledge, attitudes and experience in the care of people with dementia.

**Keywords**

Dementia; Knowledge; Attitudes; Care approach; Medical students; Nursing students

**Background**

There are more than 50 million of people living with dementia across the world and this number is expected to rise rapidly to over 152 million by 2050 [[1](#_ENREF_1)]. The total global estimated cost for dementia in 2015 was $957.56 billion USD and dementia is expected to cost $2.54 trillion USD in 2030 [[2](#_ENREF_2), [3](#_ENREF_3)]. China has the largest population of people with dementia in the world [[1](#_ENREF_1)]. The population of people with dementia in China is expected to rise dramatically from 9.5 million in 2016 to over 16 million by 2030 [[3](#_ENREF_3)]. The average cost per person with dementia per year is $19,144 USD, which far exceeds average per capita disposable income in China [[4](#_ENREF_4)].

There is an urgent need to improve the healthcare for people living with dementia around the world now and in the future. Although dementia cannot be cured, people living with dementia can live well, delay disease progression and achieve health–related quality of life when they receive a timely diagnosis, effective treatment and high-quality care [[5](#_ENREF_5)]. However, more than 50% of people living with dementia in developed countries are under-detected and under-diagnosed [[3](#_ENREF_3)]. In developing countries, including China, the diagnosis rate is only 5-10% [[3](#_ENREF_3), [6](#_ENREF_6)]. Even when people with dementia receive a diagnosis, the care provided is often uncoordinated, fragmented and unresponsive to individual needs [[3](#_ENREF_3), [7](#_ENREF_7)].

High-quality dementia care requires inter-disciplinary collaboration in order to address individual care needs associated with the condition and other comorbidities [[5](#_ENREF_5), [8](#_ENREF_8)]. Health professionals, such as registered nurses, physicians, general practitioners, neuropsychologists and geriatricians, need to work collaboratively to contribute their specialist knowledge to the diagnosis, treatment, care and palliation of people living with dementia [[5](#_ENREF_5), [9](#_ENREF_9)]. To provide best-practice dementia care, health professionals also need to engage in shared decision-making to complement their roles and foster client-centred treatment and care [[8](#_ENREF_8), [10](#_ENREF_10)].

Undergraduate medical and nursing programs, as the major providers of education for health professionals, play a crucial role in the provision of dementia education to the healthcare workforce [[11](#_ENREF_11), [12](#_ENREF_12)]. Yet, the lack of dementia education in undergraduate curricula appears to be an international issue [[13](#_ENREF_13)]. In Australia, dementia education is not included into the education curricula of 14 medical specialties [[14](#_ENREF_14)]. In the USA, over half of health professionals feel that they are not equipped with adequate education and resources to manage people with dementia as their condition progresses [[15](#_ENREF_15)]. Although the curricula of medicine and nursing undergraduate programs in China undergoes rigorous design, there is also no standard requirement for embedded or stand-alone topics specifically focusing on dementia [[16](#_ENREF_16)]. Based on the first author’s academic experience, the study of dementia care is only 2 hours and 3 hours in the nursing and medical curriculum respectively at XX (blinded for review) University, one of the participating universities in this study. Often dementia education is delivered as a ‘tick-box’ exercise or an add-on, or there is an ad hoc approach. This kind of education has little value attached and does not positively impact on students’ knowledge, attitudes and skills in providing effective care for people with dementia [[11](#_ENREF_11)].

Numerous studies have found a lack of knowledge and inappropriate attitudes towards dementia among students in health and non-health areas [[17-20](#_ENREF_17)]. In a systematic review, Ahmad et al. found that the majority of college and university students reported low understanding of risk factors and causes related to dementia [[20](#_ENREF_20)]. In a recent cross-sectional study involving 359 adolescent students, around 79% of them perceived that people with dementia demanded too much time to keep them clean, healthy and safe[[19](#_ENREF_19)]. Moreover, in another study by Tullo et al., they found a lack of a person-centred approach to dementia care in medical students [21]. For example, most participants agreed that “it is acceptable to lie to people with dementia if you think the truth might be upsetting to hear ” and “it is preferable to try to talk to a member of the family of people with dementia first, before speaking to the patients”[[21](#_ENREF_21)]. However, few studies were conducted to compare dementia care knowledge, attitudes and person-centred care approach among health professional students for the purpose of promoting inter-professional education in dementia care.

In recent years, some efforts have been made to improve dementia education for undergraduate medical and nursing students in a global context. The Higher Education Dementia Network (HEDN) in UK has developed dementia core skills education and training framework for undergraduate programs [[22](#_ENREF_22)]. The Health and Social Care Board in Northern Ireland recently designed a Dementia Learning and Development Framework to facilitate development and provision of dementia education programs [[22](#_ENREF_22)]. These initiatives are useful for supporting the development of dementia education in undergraduate programs. However, inter-disciplinary dementia education remains brief and inadequate [[8](#_ENREF_8), [23](#_ENREF_23), [24](#_ENREF_24)]. The content, implementation and assessment of inter-disciplinary dementia education remain inherently challenging [[8](#_ENREF_8), [23](#_ENREF_23), [24](#_ENREF_24)]. Identifying the current knowledge, attitudes and care approach of students from different disciplines is seen as a key component for the development of improved inter-disciplinary dementia education[[8](#_ENREF_8), [24](#_ENREF_24)].

Most of the previous studies assessing students’ dementia knowledge, attitudes and care approach were conducted in developed countries [[25](#_ENREF_25), [26](#_ENREF_26)]. There is currently little understanding of how Chinese undergraduate medical and nursing students perceive this important topic. To meet recommended clinical practice guidelines for inter-professional dementia care [[8](#_ENREF_8), [10](#_ENREF_10)], it is imperative to evaluate and compare undergraduate medical and nursing students’ dementia knowledge, attitudes and perception of care approach to provide evidence to inform reforms to inter-disciplinary dementia education undergraduate programs in China. Moreover, although studies have identified socio-demographic factors associated with dementia knowledge, attitudes and care approach in health professionals[[27](#_ENREF_27), [28](#_ENREF_28)], these factors have not been examined in a single study with undergraduate medical and nursing students. This study addressed the gap in the dementia care literature.

**Methods**

**Aims**

The aims of this study were to examine medical and nursing students’ dementia knowledge, attitudes and perceived care approach, and explore associated factors to inform inter-disciplinary dementia education curriculum design in China.

**Study design**

From January to May 2018, a cross-sectional research survey of Chinese undergraduate nursing and medical students was completed to gather self-reported data of dementia knowledge, attitudes and perception of care approach at four universities with campuses across Eastern, Western, Southern and Northern China.

**Participants**

A convenience sampling method was used in the study. All students were invited to participate in the study if they meet the following inclusion criteria: (1) enrolled in a 5-year Bachelor of Medicine Program or a 4-year Bachelor of Nursing Program in the participating universities and (2) being willing to participate in the study.

**Sample size analysis**

The sample size was based on the primary outcome, the medical and nursing students’ dementia knowledge using the Alzheimer’s Disease Knowledge Scale (ADKS). An earlier study examined a sample of students using the same instruments and reported mean knowledge scores of 20.19±3.59 [[29](#_ENREF_29)]. Using this mean score as a reference for sample size calculation with a 95% confidence interval, a beta error of 10% and an alpha of 0.05, the required sample size was calculated using this formula:$n={(\frac{Z_{1-\alpha/2}\times\sigma}{\delta})}^{2}$. A sample size of 400 was required based on this calculation. Considering a 50% return rate in survey studies, 600 participants were needed in the study.

**Procedure**

Prior to participant recruitment, permission to contact nursing and medical students was obtained from each of the four universities. Two trained research assistants were responsible for participant recruitment and questionnaire distribution at each campus. Interested students were given detailed information about the study by a trained research assistant. Students were informed about the aim, data collection, procedures, benefits, risks and confidentiality of the study via written information. Returning the completed questionnaire survey was evidence that students consented to participate in the study and written consent was not required[[30](#_ENREF_30)]. Completion of the questionnaire took about 25 minutes. Students were requested to complete the questionnaire independently and anonymously. To guarantee anonymity and confidentiality, students submitted the completed questionnaire in a closed envelope and put it inside a closed survey collection box.

**Measurements**

The students’ demographic information included age, gender, academic year, informal caregiving experience for people with dementia, clinical practicum experience in geriatrics, dementia education or training, and interest in learning more about dementia.

The Alzheimer’s Disease Knowledge Scale (ADKS) contains 30 true/false items to assess knowledge about dementia. The items cover seven domains of dementia knowledge: symptoms, risk factors, disease progression, assessment and diagnosis, caregiving, life impact, treatment and management[[29](#_ENREF_29)]. The total score ranges from 1 to 30 with higher score indicating better dementia knowledge. The reliability of the Chinese version of the ADKS was established by Wang et al in 2015 with a Cronbach’s alpha of 0.72.[[31](#_ENREF_31)]. In the current study, the ADKS showed internal consistency with a Cronbach’s alpha of 0.70.

The Dementia Care Attitude Scale (DCAS) consists of 8 items that measure students’ attitudes towards dementia[[31](#_ENREF_31)]. Each item is rated on a 5-point Likert scale from 1 (strong disagree) to 5 (strongly agree). The instrument includes two factors (labelled as ‘Heartfelt’ and ‘Heartsink’) and each factor consists of four items. The Heartfelt factor items are positively worded and the Heartsink factor items are negatively worded. A total score is calculated by reverse coding the Heartsink factor items and summing the 8 items scored in the two factors. The minimum score value is 8 and the maximum score value is 40. A higher total score indicates more positive attitudes towards dementia. The reliability of the Chinese version of the DCAS was established by Wang et al in 2015 with a Cronbach’s alpha of 0.71[[31](#_ENREF_31)]. In the current study, the DCAS showed internal consistency with a Cronbach’s alpha of 0.70.

The Approach to Advanced Dementia Care Questionnaire (ADCQ) was used to evaluate students’ perception of care approach for people with dementia[[32](#_ENREF_32)]. The choice of answers indicates either a person-centred approach or a reality-oriented approach. Person-centred approaches are scored 1 and reality-oriented approaches are scored 0. A maximum score of 13 is possible. The reliability of the Chinese version of the ADCQ was established by Lin et al in 2012 with a Cronbach’s alpha of 0.60 [[33](#_ENREF_33)]. In the current study, the ADCQ showed internal consistency with a Cronbach’s alpha of 0.62.

**Statistical analysis**

The Statistics Product and Service Solutions (SPSS) version 22.0 statistical package (IBM Corp., Armonk, New York, USA) was used for data analysis. Two research assistants checked the surveys for any missing data and entered the data into SPSS for analysis. The Kolmogorov-Smirnov test was used to determine normality of distribution of all variables. The ADKS, DCAS and ADCQ scores showed normality of distribution. An independent-sample t test, one-way ANOVA and Pearson correlation analysis were used to compare mean scores among groups. Multivariate analysis of variance (MANOVA) was used to compare scores in each content domain and total scores between medical and nursing students. Bonferroni correction was used to adjust the alpha level used to judge statistical significance. Multivariate regression models were used to identify the factors that were significantly associated with ADKS, DCAS and ADCQ scores (dependent variables). Potential factors associated with ADKS, DCAS and ADCQ scores identified in the literature and in the bivariate analysis were entered as independent variables in three separate multivariate regression models. Preliminary analysis was conducted to ensure no violation of the assumptions of normality, linearity, multi collinearity and homoscedasticity were present in the multivariate regression analysis. Statistical significance was set at p-value<0.05 in 2-tailed tests.

**Results**

**Demographics of medical and nursing students**

Of the 1200 questionnaires distributed, 85% (n=1020) were returned. Data from participants with more than 10% missing responses were excluded (n=27); the remaining 993 (526 medical students and 467 nursing students) completed questionnaires yielded a response rate of 82.3%. The mean age of the sample was 19.9±1.8 years (range: 16-25 years). Most were female (74.4%), without informal caregiving experience for people with dementia (82.1%) and had an interest in learning more about dementia (85.5%). A higher proportion of nursing students had geriatric clinical practice experience and dementia education or training than medical students (p<0.05). The demographics of medical and nursing students are summarised in Table 1.

[Insert Table 1 about here]

**Knowledge**

The overall mean knowledge score was 19.49±2.82 (ranging from 10 to 30 out of 30) and equivalent to 65% of answers correct. Medical students demonstrated higher mean scores in the ‘symptoms’ and ‘life impact’ content domains than nursing students (p<0.05). The lowest overall score was achieved on the ‘symptoms’ content domain (53%). From analysing the four items of ‘symptoms’, a majority of students (72.3% n=718) mistakenly believed that ‘tremor or shaking of the hands or arms was a common symptom in people with Alzheimer’s disease’. In addition, only 27.8% (n=276) of students responded correctly to the item that ‘in rare cases, people have recovered from Alzheimer’s disease’ and 31% (n=308) gave the correct answer to the item that ‘if trouble with memory and confused thinking appears suddenly, it is likely due to Alzheimer’s disease’. Higher correct rates were obtained in the content domains of ‘treatment and management’ (72%) and ‘life impact’ (74%). Most students knew that ‘people whose Alzheimer’s disease was not yet severe could benefit from psychotherapy for depression and anxiety’. In the ‘life impact’ domain, most students knew the statement ‘it was safe for people with Alzheimer’s disease to drive, as long as they had a companion in the car at all times’ was wrong. Moreover, in the ‘course of disease’ domain, most students knew that ‘a person with Alzheimer’s disease became increasing likely to fall down as the disease got worse’ (Table 2).

Informal caregiving experience for people with dementia was not found to be significantly related to knowledge scores (p>0.05). Knowledge scores were significantly different when related to gender, academic year, clinical practicum experience in geriatrics, dementia education or training, and interest in learning more about dementia (p<0.05). In particular, students in year three had the highest knowledge scores.

[Insert Table 2 about here]

**Attitudes**

Table 3 shows the total mean attitude score (mean=29.92, SD=3.35) and the mean scores of two factors (‘heartfelt’ and ‘heartsink’). There were statistically significant differences in the total mean attitudes score (F=18.235, p<0.01) and mean score of the ‘heartfelt’ factor (F=7.409, p<0.01) between nursing and medical students. In the ‘heartfelt’ factor, most students agreed that that ‘much could be done to improve the quality of life of people with dementia (mean=4.33, SD=0.63) and caregivers of people with dementia’ (mean=4.20, SD=0.72). In the ‘heartsink’ factor, most students disagreed that ‘patients with dementia could be a drain on resources with little positive outcome’. However, lowest scores were achieved in the items ‘the primary care team had a limited role to play in the care of people with dementia’ (mean=2.34, SD=0.87) and ‘managing dementia was more often frustrating than rewarding’ (mean=3.52, SD=0.99). In addition, students who were female, had dementia education or training, and showed interest in learning more about dementia showed statistically significant higher attitudes scores (p<0.05). There were statistically significant decreases in attitudes scores between students in year 1 and other academic years (p<0.05).

[Insert Table 3 about here]

**Care approach**

The total mean score of care approach was 5.42±2.20, indicating most students would not apply a person-centred approach in dementia care. Over 85% (n=848) of students realised that people with dementia should be allowed to freely express themselves even in seemingly meaningless behaviour. However, more than 88% of students thought that when people with dementia participated in group activities they should be told what to do and that the aim of communicating should be to help them to better handle their life situation. Moreover, in all content domains a student’s major was not related to their dementia care approach (Table 4). There were no statistically significant differences in care approach scores between student groups.

[Insert Table 4 about here]

Factors associated with **knowledge, attitudes and care approach**

The results revealed that gender, major, clinical practicum experience in geriatrics, dementia education or training, and interest in learning more about dementia were significantly associated with dementia knowledge (F=6.614, P=0.000), explaining 5% of the variance in knowledge. The multiple regression model using attitudes as the dependent variable was statistically significant and three factors were identified to be associated with attitudes (F=10.956, P=0.000), explaining 7% of the variance. These factors were gender, dementia education or training, and interest in learning more. Moreover, only gender and interest in learning more about dementia were significantly associated with the care approach (F=2.234, P=0.023), accounting for 1% of the total variance (Table 5).

[Insert Table 5 about here]

**Discussion**

A body of evidence confirms that the most effective dementia care is provided by health professionals through inter-professional collaboration [[34-36](#_ENREF_34)]. However, few inter-disciplinary studies comparing dementia knowledge, attitudes and care approach between medical and nursing students are available. In this study, self-reported dementia knowledge, attitudes and care approach among medical and nursing students were explored to determine whether students were well prepared with core dementia care competence during their formal education years. The present study is significant in contributing to a broader understanding of the topic, providing insight into current inter-disciplinary curriculum reform of medicine and nursing undergraduate programs, and may be used to improve preparation of the future health workforce.

Our study found that the knowledge scores of nursing and medical students in China are significantly lower than their counterparts in Malta and US using the ADKS [[26](#_ENREF_26), [29](#_ENREF_29)]. Possible reasons for this difference may be that dementia education is not included in the curricula of bachelor of medicine and bachelor of nursing programs in universities in China [[37](#_ENREF_37)]. In China, education sessions in gerontology for undergraduate medical and nursing students are generally covered during the third year with our results suggesting that such education has a positive influence on students’ knowledge [[38](#_ENREF_38)]. A study by [Scerri and Scerri [26]](#_ENREF_26) also found that education sessions had a positive influence on students’ knowledge about dementia. However, it should be noted that in the present study knowledge of dementia decreases after students’ clinical placements. This may suggest that theory-based ‘gerontology’ classroom study alone is insufficient and students need to be better supported to apply theory to practice [[38](#_ENREF_38)]. The lack of suitable clinical placements for medical and nursing students is one important barrier to dementia care education[[10](#_ENREF_10), [25](#_ENREF_25)]. Strategies used to support clinical preceptors to develop dementia care competence need to be considered in the Chinese context. Clinical preceptors have a responsibility to provide a supportive clinical learning environment and involve students in a team approach to improve their care practices. Effective teaching strategies, learning resources and meaningful feedback are also vitally important to improve students’ knowledge of dementia care.

The data presented here is also indicative of the lack of knowledge about dementia symptoms (correct rate 53%) and care giving issues (correct rate 62%). A similar finding was recently observed among Chinese primary health care professionals [[39](#_ENREF_39)]. Moreover, in a survey involving 280 nursing students, questions on dementia care giving had a similar correct rate (65%) [[26](#_ENREF_26)]. More education and training are needed to increase knowledge about dementia care giving, especially person-centred care which is internationally recognised as best-practice. This present study also identified that nursing students have less knowledge about dementia symptoms and life impact compared to medical students. Yet, nurses are in an ideal position to coordinate early detection and dementia management as they constitute the majority of health professionals and have more contact and closer relationships with health service recipients. A limited knowledge of symptoms and life impact among nursing students will limit their professional roles in providing dementia care services upon graduation.

Overall, medical and nursing students expressed moderately positive attitudes towards people with dementia and their family caregivers. This result is consistent with a previous study which showed nursing students demonstrate more positive attitudes scores than medical students [[26](#_ENREF_26)]. However, nursing students in our study had a low score in person-centred dementia care. These results confirm findings from previous studies in China [[33](#_ENREF_33), [39](#_ENREF_39)] and may indicate that person-centred dementia care is not well known in China. Much work needs to be done throughout China to promote the person-centred care approach as the gold standard in dementia care and to integrate this into government policies, dementia care guidelines, curricula of health professional education and clinical practice [[33](#_ENREF_33), [40](#_ENREF_40)]. It is recommended that person-centred care education be fostered by creative, experimental and reflective processes [[41](#_ENREF_41)]. Educators need to facilitate students’ learning of dementia care by creating respectful dialogue through critical thinking, self-awareness, personal knowing and reflection using case scenarios.

The present study identified that clinical practicum experience in geriatrics, dementia education and training, and interest in learning dementia care were associated with better knowledge, attitudes and care approach. This finding supports previous studies in developed countries [[26](#_ENREF_26), [42](#_ENREF_42), [43](#_ENREF_43)]. University academics, physicians, registered nurses and clinical preceptors need to work closely to involve students in dementia care to produce a better qualified healthcare workforce. The dementia care curricula need to address the full range of appropriate knowledge and skills, and to be embedded across topics. For example, ethics, communication, evidence-based science and psychology topics [[11](#_ENREF_11)]. One-off and short-term sessions, lectures or assessments that have few activities to engage students have limitations in improving students’ dementia knowledge, attitudes and care approach. Moreover, collaborative practice and teamwork among medical and nursing students are core to developing their competence in inter-disciplinary dementia care and need to be incorporated in practicum topics throughout the medical and nursing curricula in undergraduate programs [[8](#_ENREF_8), [44](#_ENREF_44)].

**Strengths and limitations of the study**

To our knowledge, this study is the first to evaluate dementia knowledge, attitudes and care approach among undergraduate medical and nursing students in China. The main strengths of the present study are the participation of four universities and the large sample size, allowing a fairly good generalisability and representativeness of the findings. The high response rate also represents students’ interest in dementia. There are several limitations to this study that should be considered. As with cross-sectional design, data are collected only once over a relative short period of time, limiting the ability to draw conclusions of possible causal relationships. Scores from self-report measures can be influenced by social desirability bias. Therefore, attitudes scores should be interpreted cautiously. In the present study, a small percentage of variance in the ADKS, DCAS and ADCQ multivariate regression models was identified. Possible reasons for these results include that the majority of participants were female and without caregiver experience, dementia education and clinical experience. Moreover, it is difficult to know how closely the sample reflects the wider cohort of medical and nursing students in China. These multivariate regression models need to be further tested in future studies.

**Conclusion**

The present study identified that medical students enrolled in a 5-year undergraduate program and nursing students enrolled in a 4-year undergraduate program in China demonstrate low scores for dementia knowledge and person-centred care approach, while showing moderately positive attitudes towards dementia. Findings have implications for curriculum intervention and further research to strengthen dementia education for medical and nursing students, especially considering the rapidly ageing population and demand for high-quality care for people with dementia. First, comprehensive undergraduate dementia care education that includes standalone dementia care topics and/or integrated dementia care content needs to be a key component in nursing and medical curricula. Second, inter-professional dementia education needs to be established in the nursing and medical curricula to enable nursing and medical students to learn and share dementia knowledge, skills and experiences. This kind of education will prepare them to provide inter-disciplinary care for people with dementia. Third, studies on effective teaching and learning strategies to improve inter-professional learning and inter-disciplinary collaboration between nursing and medical students in dementia care education are needed.

**Abbreviations**

USD, USA Dollar; HEDN, Higher Education Dementia Network; ADKS, Alzheimer’s Disease Knowledge Scale; DCAS, Dementia Care Attitude Scale; ADCQ, Approach to Advanced Dementia Care Questionnaire.

**Declarations**

**Ethics approval and consent to participate**

Ethical approval for the study was gained from Human Research Ethics Committee of Xiangya School of Nursing, Central South University [study number 2017011]. Students were informed about the aim, data collection, procedures, benefits, risks and confidentiality of the study via written information. Returning completed questionnaire survey was the evidence that students consented to participate in the study and written consent was not required.

**Consent for publication**

Not applicable

**Availability of data and material**

The datasets used and/or analysed during the current study are available from the corresponding author on reasonable request.

**Competing interests**

The authors declare that they have no competing interests.

**Funding**

This study was funded by Philosophy and Social Science Foundation of Hunan Province [Grant number: 18YBQ128], and Humanities and Social Science Foundation of Ministry of Education, People’s Republic of China [Grant number: 19YJCZH177]. The funding sources had no role in study design, data collection and analysis, decision to publish or preparation of the manuscript.

**Author’s contributions**

LDX, YW and RH designed the study. YW and RH carried out most of the data collection. YW undertook most of the data analysis and wrote the first draft of this paper. All authors contributed to data analysis, interpretation, critically commented and approved the final manuscript.

**Acknowledgements**

We are grateful to all the participants who took part in this study.

**Reference**

1. Alzheimer's Disease International: World Alzheimer report 2019: attitudes to dementia. In. London: Alzheimer's Disease International; 2019.

2. Jia J, Wei C, Chen S, Li F, Tang Y, Qin W, Zhao L, Jin H, Xu H, Wang F: The cost of Alzheimer's disease in China and re-estimation of costs worldwide. Alzheimer's & Dementia 2018, 14(4):483-491.

3. Alzheimer's Disease International: World Alzheimer Report 2016: improving healthcare for people living with dementia. In. London: Alzheimer's Disease International; 2016: 2016.

4. Jia J, Wei C, Chen S, Li F, Tang Y, Qin W, Zhao L, Jin H, Xu H, Wang F: The cost of Alzheimer's disease in China and re‐estimation of costs worldwide. Alzheimer's & Dementia 2018, 14(4):483-491.

5. Michalowsky B, Xie F, Eichler T, Hertel J, Kaczynski A, Kilimann I, Teipel S, Wucherer D, Zwingmann I, Thyrian JR: Cost-effectiveness of a collaborative dementia care management—Results of a cluster-randomized controlled trial. Alzheimer's & Dementia 2019.

6. Zhu Y, Liu H, Lu X-L, Zhang B, Weng W, Yang J, Zhang J, Dong M-J: Prevalence of dementia in the People’s Republic of China from 1985 to 2015: a systematic review and meta-regression analysis. BMC public health 2019, 19(1):578.

7. World Health Organization: Global action plan on the public health response to dementia 2017–2025. In.; 2017.

8. Cartwright J, Franklin D, Forman D, Freegard H: Promoting collaborative dementia care via online interprofessional education. Australasian journal on ageing 2015, 34(2):88-94.

9. Grand JH, Caspar S, MacDonald SW: Clinical features and multidisciplinary approaches to dementia care. Journal of multidisciplinary healthcare 2011, 4:125.

10. Surr CA, Gates C, Irving D, Oyebode J, Smith SJ, Parveen S, Drury M, Dennison A: Effective dementia education and training for the health and social care workforce: a systematic review of the literature. Review of educational research 2017, 87(5):966-1002.

11. Collier E, Knifton C, Surr C: Dementia education in higher education institutions. In.: Elsevier; 2015.

12. Downs M, Capstick A, Baldwin PC, Surr C, Bruce E: The role of higher education in transforming the quality of dementia care: Dementia studies at the University of Bradford. International psychogeriatrics 2009, 21(S1):S3-S15.

13. Hvalič-Touzery S, Skela-Savič B, Macrae R, Jack-Waugh A, Tolson D, Hellström A, de Abreu W, Pesjak K: The provision of accredited higher education on dementia in six European countries: An exploratory study. Nurse education today 2018, 60:161-169.

14. Ibrahim JE, Davis M-C: Availability of education and training for medical specialists about the impact of dementia on comorbid disease management. Educational Gerontology 2013, 39(12):925-941.

15. Adler G, Lawrence BM, Ounpraseuth ST, Asghar-Ali AA: A survey on dementia training needs among staff at community-based outpatient clinics. Educational Gerontology 2015, 41(12):903-915.

16. Wang Y, Xiao LD, Ullah S, He GP, De BA: Evaluation of a nurse-led dementia education and knowledge translation programme in primary care: A cluster randomized controlled trial. Nurse Education Today 2017, 49(Complete):1-7.

17. Kwon M-S, Lee J-H: Analysis of knowledge, attitude and service requirements about dementia among nursing students. Journal of the Korea Academia-Industrial cooperation Society 2017, 18(9):177-185.

18. Scott TL, Kugelman M, Tulloch K: How medical professional students view older people with dementia: Implications for education and practice. PloS one 2019, 14(11):e0225329.

19. Isaac MG, Isaac MM, Farina N, Tabet N: Knowledge and attitudes towards dementia in adolescent students. Journal of Mental Health 2017, 26(5):419-425.

20. Ahmad Basri MAF, Subramaniam P, Ghazali SE, Singh DKA: A Review of Knowledge and Attitudes Towards Dementia Among College and University Students. Journal of Clinical & Diagnostic Research 2017, 11(11).

21. Tullo ES, Young TJ, Lee RP: Medical students’ views about person-centred communication in dementia care. Dementia 2018, 17(5):573-584.

22. Knifton C, MacRae R, Jack-Waugh A, Brown M, Surr C, Thompson R, Baillie L: Perspectives: Dementia education in Higher Education Institutions, now and in the future: the role of the professional regulatory bodies in the UK. Journal of Research in Nursing 2019, 24(3-4):271-278.

23. Mastel-Smith B, Kimzey M, Garner J, Shoair OA, Stocks E, Wallace T: Dementia care boot camp: interprofessional education for healthcare students. Journal of Interprofessional Care 2019:1-13.

24. Annear MJ, Goldberg LR, Lo A, Robinson A: Interprofessional curriculum development achieves results: Initial evidence from a dementia-care protocol. Journal of Interprofessional Care 2016, 30(3):391-393.

25. Robinson A, Cubit K: Caring for older people with dementia in residential care: nursing students’ experiences. Journal of Advanced Nursing 2007, 59(3):255-263.

26. Scerri A, Scerri C: Nursing students' knowledge and attitudes towards dementia — A questionnaire survey. Nurse Education Today 2013, 33(9):962-968.

27. Keogh B, To WT, Daly L, Hynes G, Kennelly S, Lawlor B, Timmons S, O’Reilly S, Bracken-Scally M, Ciblis A: Acute hospital staff’s attitudes towards dementia and perceived dementia knowledge: a cross-sectional survey in Ireland. 2020.

28. Wang Y, Xiao LD, Luo Y, Xiao S-Y, Whitehead C, Davies O: Community health professionals’ dementia knowledge, attitudes and care approach: a cross-sectional survey in Changsha, China. BMC geriatrics 2018, 18(1):122.

29. Carpenter BD, Steve B, Otilingam PG, Hanson PK, Margaret G: The Alzheimer's Disease Knowledge Scale: development and psychometric properties. Gerontologist 2009, 49(2):236.

30. Polit DF, Beck CT: Essentials of nursing research: Appraising evidence for nursing practice: Lippincott Williams & Wilkins; 2009.

31. Wang Y, Xiao LD, He G-P: A comprehensive approach to psychometric assessment of instruments used in dementia educational interventions for health professionals: a cross-sectional study. International journal of nursing studies 2015, 52(2):568-577.

32. Normann HK, Asplund K, Norberg A: Attitudes of registered nurses towards patients with severe dementia. Journal of clinical nursing 1999, 8(4):353-359.

33. Lin P-C, Hsieh M-H, Lin L-C: Hospital nurse knowledge of and approach to dementia care. Journal of Nursing Research 2012, 20(3):197-207.

34. Heeren M-J, Langelaan M, de Jong C, de Groot JF: Inter-professional collaboration and communication during acute hospitalization of older patients with dementia. International Journal of Integrated Care (IJIC) 2018, 18.

35. Jackson M, Pelone F, Reeves S, Hassenkamp AM, Emery C, Titmarsh K, Greenwood N: Interprofessional education in the care of people diagnosed with dementia and their carers: a systematic review. BMJ open 2016, 6(8):e010948.

36. Tan ZS, Damron‐Rodriguez J, Cadogan M, Gans D, Price RM, Merkin SS, Jennings L, Schickedanz H, Shimomura S, Osterweil D: Team‐based interprofessional competency training for dementia screening and management. Journal of the American Geriatrics Society 2017, 65(1):207-211.

37. Chen Z, Yang X, Song Y, Song B, Zhang Y, Liu J, Wang Q, Yu J: Challenges of dementia care in China. Geriatrics 2017, 2(1):7.

38. Shen J, Xiao LD: Factors affecting nursing students' intention to work with older people in China. Nurse education today 2012, 32(3):219-223.

39. Wang Y, Xiao LD, Luo Y, Xiao SY, Whitehead C, Davies O: Community health professionals' dementia knowledge, attitudes and care approach: a cross-sectional survey in Changsha, China. Bmc Geriatrics 2018, 18(1):122.

40. Chenoweth L, King MT, Jeon Y-H, Brodaty H, Stein-Parbury J, Norman R, Haas M, Luscombe G: Caring for Aged Dementia Care Resident Study (CADRES) of person-centred care, dementia-care mapping, and usual care in dementia: a cluster-randomised trial. The Lancet Neurology 2009, 8(4):317-325.

41. Schwind JK, Lindsay GM, Sue C, Debbie M, Barb M: Opening the black-box of person-centred care: An arts-informed narrative inquiry into mental health education and practice. Nurse Education Today 2014, 34(8):1167-1171.

42. Smyth, Wendy, Fielding, Elaine, Beattie, Elizabeth, Franklin, Sara, MacAndrew, Margaret: A survey-based study of knowledge of Alzheimer's disease among health;care staff. Bmc Geriatrics 2013, 13(1):1-8.

43. Travers CM, Beattie E, Martin-Khan M, Fielding E: A survey of the Queensland healthcare workforce: attitudes towards dementia care and training. BMC geriatrics 2013, 13(1):101.

44. Nicol P: Interprofessional education for health professionals in Western Australia: perspectives and activity. Sydney: University of Technology Sydney 2013.
