# Supplementary material for: A comparative study of dementia knowledge, attitudes and care approach among Chinese nursing and medical students
Source: BMC Med Educ. 2020 Nov 16;20:436. doi: 10.1186/s12909-020-02365-1 (PMC7670709; doi:10.1186/s12909-020-02365-1)
Supplement: Supplementary file 1 — Additional file 1. [file 12909_2020_2365_MOESM1_ESM.doc]

09 June 2020

The Editor

BMC Medical Education

Dear Editor,

I am writing to you to submit a manuscript titled ‘A comparative study of dementia knowledge, attitudes and care approach among Chinese nursing and medical students’ to the BMC Medical Education for consideration of publication. This is a collaborative project between Central South University (CSU) in China and Flinders University (FU) in Australia. Therefore, we have proposed Associate Professor Yao WANG (CSU) and Professor Lily Dongxia XIAO (FU) as corresponding authors.

Dementia has been recognised as a public health priority by WHO. Dementia care requires inter-disciplinary collaboration starting from formal health professional education. Yet, little is known about how undergraduate medical and nursing students perceive dementia care in China, which has the largest population of people with dementia in the world. The present study is significant in contributing to a broader understanding of the topic, providing insight into current inter-disciplinary curriculum reform of medicine and nursing undergraduate programs, and may be used to improve preparation of the future health workforce.

In the present manuscript, all authors have met the criteria for authorship. All the authors have reviewed this manuscript and agree with the final version. On behalf of all the authors, I declare that the work is original research and has not been published and is not being considered for publication elsewhere, in whole or in part.

We appreciate your support during the review period.

Yours Sincerely

Dr Yao WANG on behalf of the authors
