# Supplementary material for: A comparative study of dementia knowledge, attitudes and care approach among Chinese nursing and medical students
Source: BMC Med Educ. 2020 Nov 16;20:436. doi: 10.1186/s12909-020-02365-1 (PMC7670709; doi:10.1186/s12909-020-02365-1)
Supplement: Supplementary file 2 — Additional file 2. [file 12909_2020_2365_MOESM2_ESM.doc]

**Response to Reviewers**

| **Reviewer 1** | **Comments** | **Responses** |
| --- | --- | --- |
| 1 | The authors have answered all the comments, but they still have to add the Cronbach alpha scores for their measures in the current study. | Thanks for your comment. We have added the Cronbach alpha scores for measurements in the current study.  Please check the Measurements section (Page 10-11). |
